# F-Net: Follicles Net an efficient tool for the diagnosis of polycystic ovarian syndrome using deep learning techniques

**DOI:** 10.1371/journal.pone.0307571

**Published:** 2024-08-15

**Authors:** Sowmiya S., Snekhalatha Umapathy, Omar Alhajlah, Fadiyah Almutairi, Shabnam Aslam, Ahalya R. K.

**Affiliations:** 1 Biomedical Engineering Department, Faculty of Engineering and Technology, SRM Institute of Science and Technology, Chennai, Tamil Nadu, India; 2 Department of Applied Computer Sciences, Applied Computer Science College, King Saud University, Riyadh, Saudi Arabia; 3 Department of Information System, College of Computer and Information Sciences (CCIS), Majmaah University, Al Majmaah, Saudi Arabia; 4 Department of Information Technology, College of Computer and Information Sciences (CCIS), Majmaah University, Al Majmaah, Saudi Arabia; 5 Department of Biomedical Engineering, Easwari Engineering College, Chennai, Tamil Nadu, India; UFRN: Universidade Federal do Rio Grande do Norte, BRAZIL

## Abstract

The study’s primary objectives encompass the following: (i) To implement the object detection of ovarian follicles using you only look once (YOLO)v8 and subsequently segment the identified follicles using a hybrid fuzzy c-means-based active contour technique. (ii) To extract statistical features and evaluate the effectiveness of both machine learning (ML) and deep learning (DL) classifiers in detecting polycystic ovary syndrome (PCOS). The research involved a two different dataset in which dataset1 comprising both normal (N = 50) and PCOS (N = 50) subjects, dataset 2 consists of 100 normal and 100 PCOS affected subjects for classification. The YOLOv8 method was employed for follicle detection, whereas statistical features were derived using Gray-level co-occurrence matrices (GLCM). For PCOS classification, various ML models such as Random Forest (RF), *k*- star, and stochastic gradient descent (SGD) were employed. Additionally, pre-trained models such as MobileNet, ResNet152V2, and DenseNet121 and Vision transformer were applied for the categorization of PCOS and healthy controls. Furthermore, a custom model named Follicles Net (F-Net) was developed to enhance the performance and accuracy in PCOS classification. Remarkably, the F-Net model outperformed among all ML and DL classifiers, achieving an impressive classification accuracy of 95% for dataset1 and 97.5% for dataset2 respectively in detecting PCOS. Consequently, the custom F-Net model holds significant potential as an effective automated diagnostic tool for distinguishing between normal and PCOS.

## Introduction

Polycystic Ovary Syndrome (PCOS), characterized by hormonal imbalances, can impact any woman post-puberty [1] In 1953, American Gynaecologist Stein Leventhal first reported this syndrome, establishing a connection between ovarian cysts and anovulation [2]. In Worldwide, about 4% to 20% of women have been reported as PCOS [3]. As per Rotterdam’s criteria [4], the prevalence rate of PCOS in India was reported to be 10%. A small, round shape sac filled with fluid called follicles, presents over the ovary, stops producing eggs and causes infertility. Symptoms of PCOS includes irregular periods, hair fall, obesity, and facial acne.

Ultrasound plays a crucial role in the diagnosis of follicles [5]. This non-invasive and cost-effective technique is employed for both diagnostic and therapeutic purposes. Increased insulin and testosterone levels in PCOS affected women contribute to elevated risk of cancer cell growth [6]. Therefore, early diagnosis of PCOS is crucial. Clinicians face a challenge in manually diagnosing the size of each follicle, which falls within the range of 2-9mm. Moreover, follicles smaller than 2mm are disregarded due to the high speckle noise present in ultrasound images [7]. Based on recent research, PCOS manifests with diverse symptoms, leading clinicians to recommend various diagnostic tests, such as ultrasound, blood tests, and radiography [8]. The results of these tests take a longer time, and the process of diagnosing clinical images is laborious. Therefore, we have developed a new method to address these limitations by incorporating an artificial intelligence (AI)-based custom convolutional neural network (CNN) for the early identification of PCOS.

In recent times, the field of AI has seen swift advancements in detecting PCOS, driven by the application of machine learning (ML) and deep learning (DL) techniques [9, 10]. Nilofer et al. employed a cluster-based segmentation approach to segment the follicles, conducted statistical feature extraction methods, and employed a novel hybrid ML method for classification [11]. The authors attained a classification accuracy of 97% by employing the Improved Fruit Fly Optimization–Artificial Neural Network (IFFOA-ANN). Sumathi et al. performed segmentation, feature extraction, and utilized CNN classification to identify cysts [12]. Watershed segmentation was employed to segment the regional cyst. Essential parameters from the segmented region, such as area and perimeter, were measured for clinical analysis to determine the cyst type, size, and location. The trained CNN yielded an accuracy of 85%.

Kumar et al. developed a distinctive segmentation method known as the Improved Chan Vase (ICV) segmentation method [13]. ICV method achieved the desired outcome with reduced iterations and learning time. These results can be employed for extracting features and classifying PCOS ultrasound images. Gopala et al. segmented the follicle from the ovary using Otsu method and extracted features using a hybrid GIST-MDR (Global image descript—Multifactor dimensionality reduction) method [14]. The Support vector machine (SVM) achieved an improved classification accuracy of 93.82%, while linear discriminant analysis (LDA) attained 91.05%. Additionally, Random Forest (RF), and Naïve Bayes (NB) achieved accuracy rates of 89.7%. and 88.26%, respectively. Rachana et al implemented the Otsu method for follicle segmentation. In their study, they employed *k*-NN supervised classification and compared it with other classifiers [15]. Remarkably, *k*-NN surpassed the performance of other classifiers, achieving an excellent accuracy as 97%. Deshpande and colleagues utilized multiscale morphological feature extraction along with an SVM classifier, followed by segmentation in their study [16]. They integrated biological factors such as hormone levels, body mass index (BMI), menstrual cycle duration, and the follicles count observed in ultrasound images for classification purposes. Remarkably, their proposed method attained an accuracy rate of 95%.

Mehr et al conducted a study on the early diagnosis of PCOS utilizing the Kaggle dataset. They used demographic and other clinical features as input for various ML classifiers [17]. They implemented ensemble RF, adaboost and multi-layer perceptron for the categorization of PCOS and normal. Srinithi et al., performed automated detection of PCOS using ML techniques [18]. The authors used logistic regression, *k*-NN and RF regression for classification and yielded the accuracy of 89%, 86% and 87% respectively. They achieved the accuracy of 93.33% for CNN. Elmannai et al used various ML classifiers such as decision tree (DT), NB, *k*-NN, SVM, XGBoost, RF and adaboost for the detection of PCOS [19]. They used demographic and clinical features as input. They also compared the stacking ML with RF classifier and attained excellent accuracy. Lv et al detected PCOS from the scleral images [20]. They segmented the scleral image using attention U-Net. They extracted the automated features from the scleral images using ResNet 18, VGG16, VGG19 and Inception V3 model. The ResNet 18 outperformed with the detection accuracy of 92.9% compared to other networks. They used multi-instance model to combine all the features vectors, which were then input into a multi-layer perceptron for the ultimate classification of PCOS and normal cases.

Most of the existing literature relies on ML algorithms that necessitate segmentation and manual feature extraction methods, leading to a significant time investment. Furthermore, pre-trained models trained on extensive ImageNet datasets often exhibit subpar performance when employed in PCOS classification tasks. The proposed study aims to establish an automated system for the detection of PCOS by integrating ML and DL techniques, complemented by object detection methods. The objectives include:—(i) To detect the follicles using You Only Look Once (YOLO)v8 deep learning model and to segment the detected follicles using Fuzzy c-means clustering-based active contour, and subsequent classification of the PCOS and normal based on ML approach (ii) To evaluate the performance of customized FollicleNet (FNet) model, pre-trained classifiers, and ML algorithms in the classification of PCOS and non-PCOS.

The main contribution of the study was as follows:

- The object detection method ‘named’ YOLOv8 deep learning model was used for automated identification of follicles in PCOS.
- The segmentation of detected follicles was performed using hybrid algorithm named Fuzzy c-means clustering-based active contour.
- A customized Follicle Net (F-Net) model was developed for automated classification of PCOS and normal and compared its performance with state of art methods like Vision transformer.

The paper is organized as follows: The methods section presents a comprehensive methodology for object detection, including segmentation through the fuzzy c-means active contour method, and classification using a custom F-Net in comparison to pre-trained models and Vision transformer. The result section demonstrates the detection and segmentation of follicles in PCOS, followed by feature extraction and classification using machine learning and deep learning techniques. The discussion section provides an in-depth discussion about the results in the proposed work compared to related literature works and concludes the paper.

## Methodology

### Data collection

**Fig 1:**
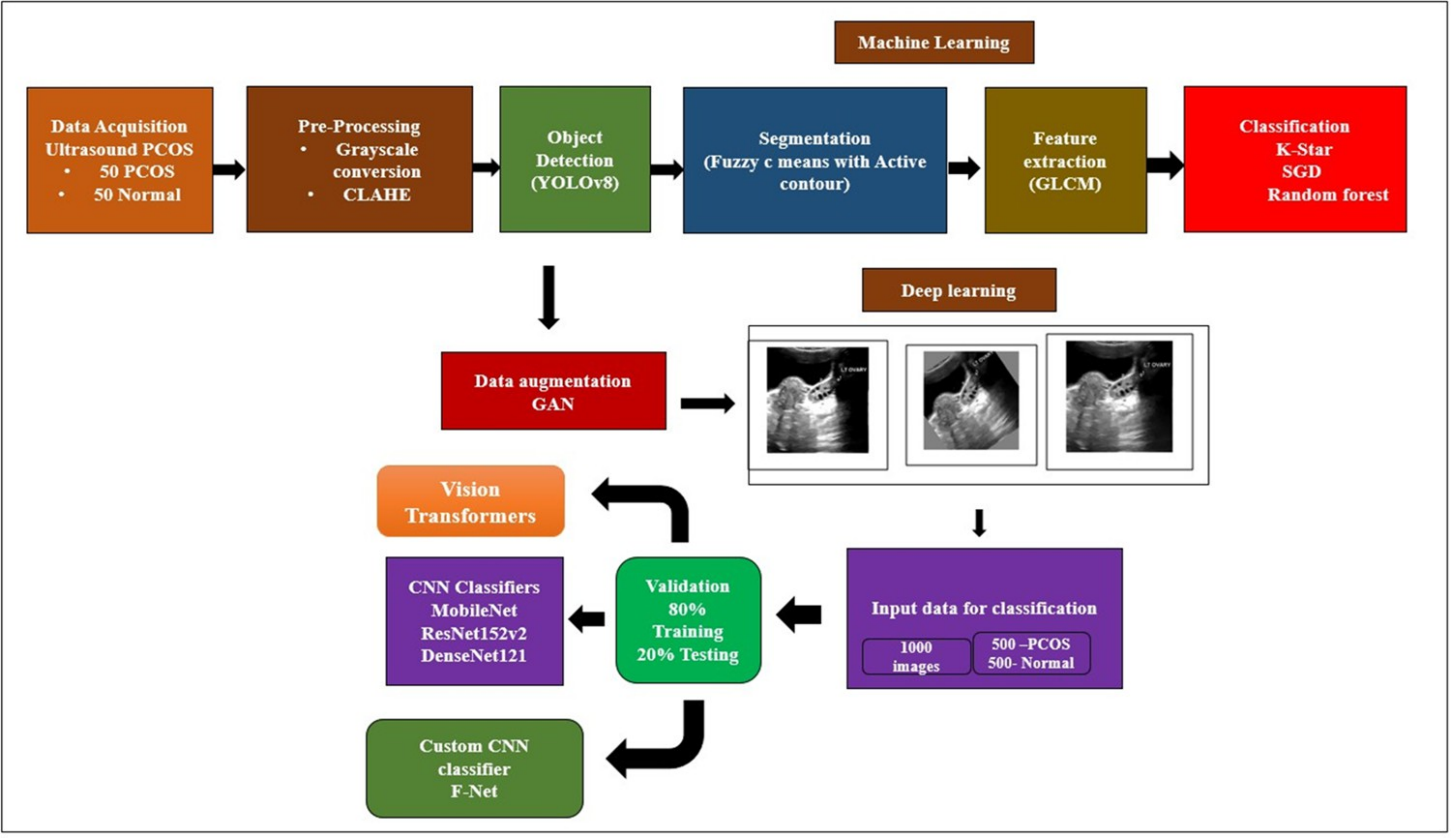
Schematic overview of the outlined research in PCOS detection.

In our study, the real-time data was collected from SRM Medical College, Hospital and Research Centre (SRMCH & RC), Kattankulathur, Tamil Nadu, India. The dataset1 consists of abdominal ultrasound images from a total of 100 subjects, 50 individuals with confirmed PCOS and 50 individuals without the condition. The dataset2 consists of ultrasound images from a total of 200 subjects, 100 subjects with confirmed PCOS and 100 Normal collected from the SRMCH&RC during the month of March and April 2024. The study comprising includes subjects aged between 18- and 45-years encompassing patients diagnosed with multiple follicles in the ovary and irregular periods, as well as healthy volunteers. All subjects participating in the study provided a written informed consent form. The study received approval from the SRM Institute ethics committee, with the ethical clearance number being SRMIEC-ST0922-203. The manual annotation has been performed by experienced Sonologist and has been confirmed with other sonologists in the Hospital. Hence based on their confirmation, ground truth segmentation was carried out. However, even when manual annotation still be time-consuming, accurate results can be obtained. Fig 1 illustrate the schematic representation of the outlined research on PCOS detection.

### Pre-processing

Initially, the images are converted to grayscale to eliminate colour and water content, ensuring the preservation of brightness and contrast. Due to the typically intense presence of speckle noise in ultrasound images, critical information in the ultrasound image is often obscured. Therefore, denoising is a crucial step to enhance image quality and generate accurate results. In this research, the denoising process incorporated image enhancement through Contrast Limited Adaptive Histogram Equalization (CLAHE). Instead of analysing the entire image simultaneously, CLAHE operates on distinct segments of an image referred to as tiles. It blends neighbouring tiles using bilinear interpolation, successfully removing false edges. This method works effectively for improving the image’s contrast.

### Follicle detection using YOLOv8

Object detection enact a significant role in computer graphics. The proposed work implemented the object detection method using YOLO on the input image to detect the follicles. YOLO is proficient in detecting the region of interest (ROI) and is excellent in speed and accuracy [21]. The YOLO approach employs a deep CNN to recognize objects when provided with an image as input. The CNN architecture serves as the basis for YOLO [22]. The initial 20 convolution layers are pre-trained using the ImageNet dataset. In the proposed study, an average pooling layer and a fully connected layer were added to facilitate the detection of follicles. Previous research has shown that incorporating convolutional and connected layers into a pre-trained network can enhance network performance and the effectiveness of object detection tasks. In YOLO, the final fully connected layer is responsible for predicting the target class and bounding box coordinates. The input image is partitioned into an S x S grid, and a follicle is identified by a square box if its origin lies within that box. Consequently, each square box provides projections of bounding boxes along with their associated accuracy values. These accuracy values signify the extent to which the bounding boxes accurately predict the follicles in the ultrasound image. YOLO predicts multiple bounding boxes for each square box. During training, only one bounding box predictor needs to be assigned to each follicle. Using YOLO, the predictor discerns a follicle by identifying the prediction with the highest Intersection over Union (IOU) compared to the actual data. This process leads to the bounding box predictors becoming more refined and specific. Each predictor has been enhanced by projecting specific sizes, aspect ratios, or object classifications, contributing to an overall improvement in the total recall score. Non-maximum suppression (NMS) plays a crucial role in YOLO models, serving as a post-processing procedure that enhances the speed and accuracy of object detection. In object detection, it is common to generate multiple bounding boxes for a single object in an image. These bounding boxes may depict the same entity, even if they overlap or are positioned in various parts of the image. NMS is applied to identify and eliminate redundant or imprecise bounding boxes from images, resulting in a single bounding box for each item within the image.

The latest iteration of Ultralytics’ YOLO object detection and segmentation technology is YOLOv8. Representing a cutting-edge and state-of-the-art (SOTA) model, YOLOv8 enhances the performance of its predecessors by incorporating new features, thereby improving efficiency and adaptability. YOLOv8 introduces several notable advancements, including a backbone network, an anchor-free detection head, and a loss function. Its adaptability renders it a compelling option for various tasks, such as object identification and image segmentation. Notably, YOLOv8 demonstrates high efficiency and compatibility with various hardware platforms, including CPUs and GPUs [23]. The architecture of YOLOv8 is illustrated in the S1 Fig.

### Segmentation using Fuzzy-c-means based active contour method

In the proposed study, segmentation is performed using a hybrid algorithm known as the Fuzzy-c-means (FCM) based active contour method. FCM, an unsupervised ML technique, is employed to partition the data into various groups or clusters. This division ensures that data points within the same group are similar, while those in distinct groups exhibit significant differences [24].

In FCM, a dataset is divided into n groups, and each data object is assigned a degree of membership in each cluster, ranging from 0 to 1. The sum of membership values for each data point adds up to one. The FCM goes through two iterative stages to reach a solution. In the initial stage, each data object is allocated membership values for each cluster, and in the subsequent stage, the data item is assigned to the group with the highest membership value [25]. FCM algorithm is described as follows: -

- Randomly select ‘n’ number of clusters
- Fix the initial membership matrix.
- Distribute the number of clusters.
- Stop the iteration if the minimum value is achieved.

$$J(U,V)=\sum_{i=1}^{n}\sum_{j=1}^{c}{\left(\mu_{i,j}\right)}^{m}{\left|x_{i-}v_{j}\right|}^{2}$$ (1)

In Eq (1), the objective function is denoted as J, the number of clusters is represented by c, and the membership of the i^th^ data to the j^th^ cluster center is assigned as μ_ij_ [26].

Active contour is a segmentation technique that employs energy forces and constraints to extract key pixels from an image. The snake model is a method capable of addressing a variety of segmentation challenges. Its primary goal is to locate and define the target object for segmentation, utilizing prior information, especially for complex objects, about the contour of the target object. Active snake models, commonly referred to as snakes, are typically configured by applying splines focused on minimizing energy and subsequently applying various forces that govern the image [27]. MATLAB software version R2022a (Mathworks, USA) is used for pre-processing, segmentation, and feature extraction.

### Feature extraction

From the segmented images, ten spatial and textural elements were extracted using Gray level co-occurrence matrix (GLCM). These characteristics include homogeneity, mean, variance, standard deviation, entropy, contrast, energy, correlation, skewness, and kurtosis, as described in Eqs (2) to (7).

The mean is calculated as the sum of all pixel values divided by the image size. Standard deviation quantifies the dispersion of the image, while variance is the square of the standard deviation. Entropy serves as a measure of the irregularity within the cyst region.

Contrast *-*Measures the amount of color or gray image difference in the region.


$$f_{1}=\sum_{m=0}^{N_{g-1}}m^{2}\{\sum_{i=1}^{N_{g}}\sum_{j=1}^{N_{g}}\rho d,v^{0(i,j)}\},|i-j|=m$$ (2)

Correlation–Measure the linearity of the neighboring pixels

$$f_{2}=\frac{\sum_{i=1}^{N_{g}}\sum_{j=1}^{N_{g}}(ij)\rho ,v^{0}(i,j)-\mu_{x}\mu_{y}}{\sigma_{x}\sigma_{y}}$$ (3)

Energy–Measure the uniformity of the segmented cyst image region.


$$f_{3}=-\sum_{i=1}^{N_{g}}\sum_{j=1}^{N_{g}}\rho^{2}d,v^{0(i,j)}$$ (4)

Homogeneity–measures the similar characteristics of the region.


$$f_{4}=\sum_{i=1}^{N_{g}}\sum_{j=1}^{N_{g}}\frac{\rho d,v^{0(i,j)}}{1+{\left(i-j\right)}^{2}}$$ (5)

Skewness—Measures the random variable’s probability distribution that deviates from the normal distribution.


$$f_{5}=\frac{1}{N}\sum_{i=1}^{N}{\left[\frac{\left(x_{i}-\bar{x}\right)}{\sigma }\right]}^{3}$$ (6)

Kurtosis–Measure of peak distributions of the potential values in each pixel

$$f_{6}=\frac{1}{N}\sum_{i=1}^{N}{\left[\frac{\left(x_{i}-\bar{x}\right)}{\sigma }\right]}^{4}$$ (7)

### Machine learning classification

#### *k*-star classifier

The *k*-star algorithm is a graph and statistics-based approach utilizing *k*-NN method. In the k-star classifier, n data points are categorized into k groups, and each data point is associated with the cluster having the closest mean. Described as an instance-based learner, the *k*-star algorithm employs entropy to compute proximity. Notably, it is a standardized method adept at handling missing values, real-valued attributes, and symbolic attributes [28].

A set of data points, denoted as n, is assigned to the most probable class, yi, where m equals 1. *k* * is described in Eq (8) as given below:

$$k*(y_{i},m)=lnP(y_{i},m)$$ (8)

P represents the chances of x reaching y via odd path. It outlines real-valued characteristics, symbolic features, and lacking values consistently. Training requires a significant amount of time [29].

#### Stochastic gradient descent (SGD)

SGD is a ML algorithm aligned with the SVM model. Known for its simplicity, efficiency, and reliability, SGD is versatile, addressing regression problems and classifications. It enhances classification prediction accuracy by adjusting model parameters to best fit the provided data. This adjustment involves updating the model coefficients and calculating the error using only a random subset of training examples at each iteration. This characteristic allows for swift training of the data, minimizing computational workload in each iteration [30]. Notably, the classifiers within this module are adapt at handling issues involving over 100,000 training examples and more than 10,000 features [31]. The hyperparameter used in SGD are learning rate (0.001) and estimators (100).

#### Random Forest classifier

RF classifier falls under the category of decision tree classifiers, operating as a meta-estimator. It aligns with various nodes in the dataset, balancing them to enhance prediction accuracy while preventing overfitting. Node size control is achieved by manipulating the number of samples, utilizing bootstrap (set to true), and initializing the tree-building process with the dataset. The construction of the RF classifier is grounded in adaptive tree learning, where both the root and leaf trees contribute to the creation of individual tree values. The dataset consists of numerous cases serving as a training set, and N sample cases are randomly drawn from the original datasets, functioning as training datasets for tree growth. If the dataset has X input variables, a value below X indicates that each node is randomly selected. Throughout the classification of each segment, the value of X remains constant in the training dataset of N samples [32].

### Deep learning classification

#### Data augmentation using Generative Adversarial Networks (GAN)

**Fig 2:**
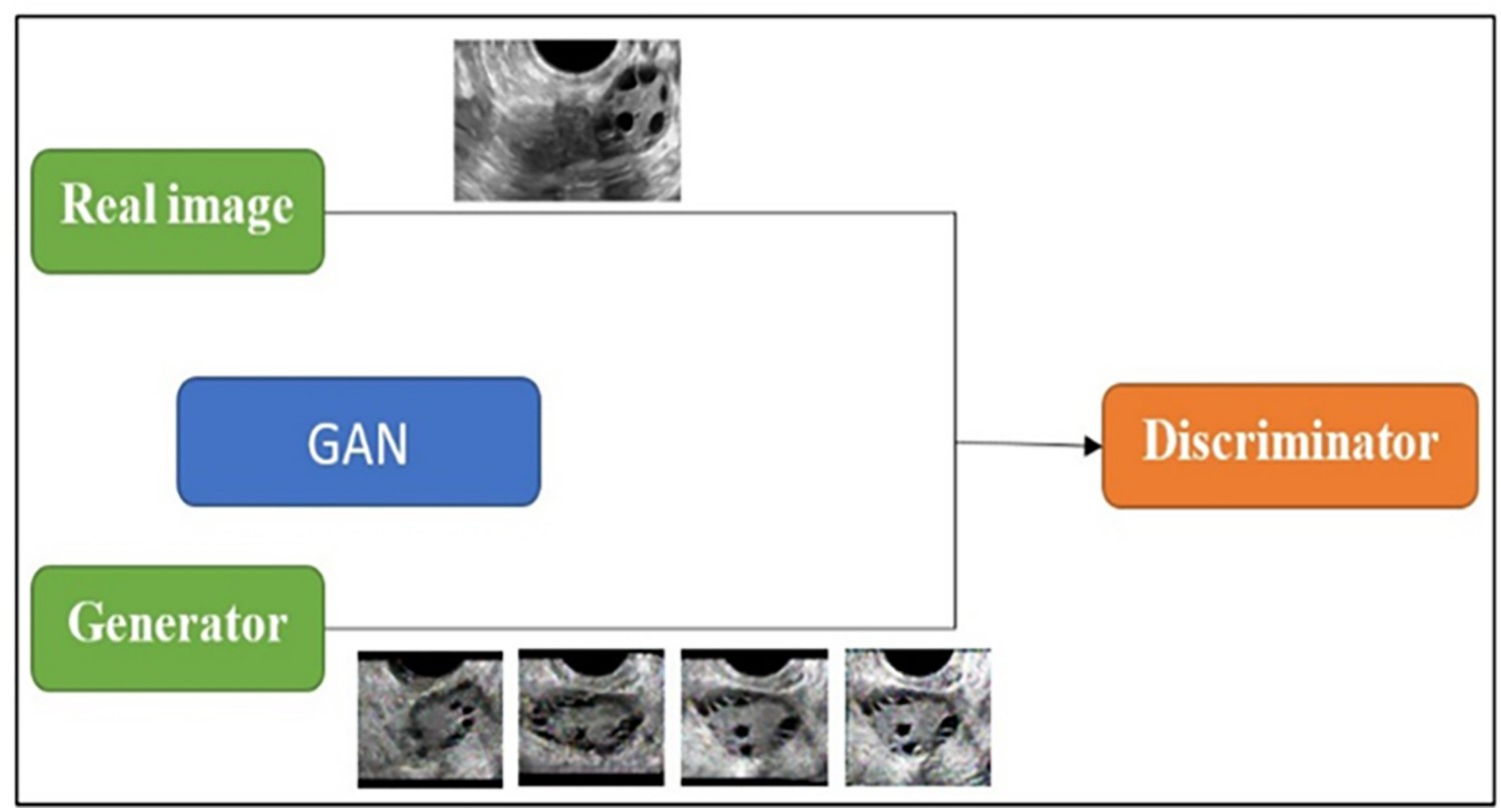
GAN for data augmentation of PCOS and Non PCOS images.

Recognizing the need for extensive datasets in deep learning models, the examined research augmented the quantity of images by employing the GAN data augmentation technique. GAN comprises two neural networks, namely the discriminator and the generator. The generator deceives the discriminator into generating similar data as that from the training set. Given the constraint of a limited real-time dataset, the dataset was expanded to 1000 using GAN. In this study, each training phase spanned 10,000 epochs, and the last 500 generated images for both PCOS and normal cases were utilized for classification. The training process began by training the discriminator with real PCOS and normal images. Subsequently, the generator produced synthetic PCOS and normal images. The discriminator was then trained with the generated data from the generator. Following this, the generator underwent training with the images generated by the discriminator. This collaborative process resulted in the creation of a new set of extensive datasets. **Fig 2** depicts the GAN diagram for the augmentation of PCOS images.

#### MobileNet

Mobile Net represents a depth-wise separable convolution technique utilized for constructing a lightweight deep convolutional network, contributing to a reduction in the number of factors when compared to conventional CNN. The creation of this convolution involves two processes: depth-wise separable convolution and point-wise convolution. The depth-wise convolution filter operates a single convolution on each input channel. The results of 1x1 filter and depth-wise convolution are linearly combined by the point-wise convolution filter. For input, the image is resized to 224x224x3 and fed into the Mobile Net model, which consists of 28 convolution layers, thirteen depth-wise convolution layers (3x3), one average pooling layer (3x3), one fully connected layer (3x3), and one SoftMax layer. Batch normalization and ReLU activation are applied after each layer [33]. Two hyperparameters, namely the width multiplier (α) and resolution-wise multiplier, are incorporated to reduce computational costs. The width multiplier scales the input and output channels, effectively reducing the network size. The alpha values range from 0 to 1, where an alpha of 1 represents a baseline MobileNet, and values less than one indicate a reduced MobileNet. The resolution multiplier (ρ) is multiplied by the feature map input, contributing to computing cost expressions that involve both width and resolution multipliers. DL techniques have been applied in a Google Colab notebook, a cloud-based platform that supports multiple programming languages. Specifically, for this work, Python version 3.9 was utilized to implement various deep learning classification techniques.

#### ResNet152V2

The Residual Network, commonly known as ResNet, is structured with more than hundreds of layers. In deep neural networks, augmenting the number of layers tends to improve performance accuracy. ResNet introduces a residual learning unit to counteract the diminishing effectiveness of deep neural networks. It operates as a feed-forward network incorporating a shortcut link that adds supplementary inputs, yielding new outputs, and thereby enhancing classification accuracy without a proportional increase in model complexity [34]. The image input size is adjusted to 224x224x3. ResNet152V2, a specific instance of ResNet, comprises 152 convolutional layers [35], a Global average pooling layer, a dense layer with 64 neurons, and a dense layer with SoftMax followed by a convolution layer [36]. Notably, ResNet152V2 and ResNet152V1 exhibit fundamental differences [37], primarily due to the incorporation of batch normalization before each weighted layer in ResNet152V2.

#### DenseNet121

A key feature of DenseNet is their ability to facilitate deeper learning in deep neural networks, with shortcut connections enhancing training proficiency. The DenseNet121 model is composed of 120 convolution layers, four average pooling layers, and one fully connected (FC) layer, with an input image size of 224x224x3. The layer configuration initiates with 64 filters of size 7x7 convolution, followed by a max-pooling layer with a stride of 2. After pooling, Dense Block (DB) 1 is formed with two convolutions (1x1, 3x3), repeated six times. Each Transition layer follows a Dense Block, comprising one convolution and one average pooling with a stride of 2. DB2 is iterated 12 times, followed by a Transition layer (TL2). Simultaneously, DB 3 consists of two convolution layers and repeats this pattern 24 times, succeeded by TL3. Proceeding to DB4, it is constructed with two convolution layers and repeats itself 16 times. In the classification phase, the network’s feature mappings are input into the classification layer, which incorporates global pooling with dimensions of 7x7. The final layer is a FC layer with two neurons and a SoftMax activation function, designed to classify between PCOS and normal conditions [38].

#### F-Net

**Fig 3:**
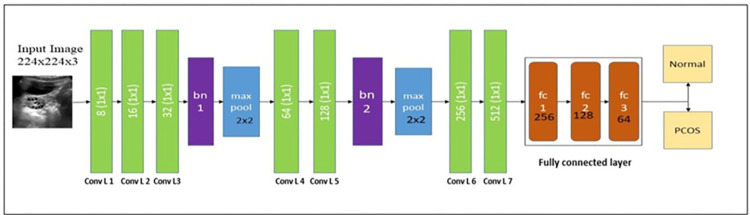
F-Net architecture for the detection of PCOS.

Nonetheless, the utilization of pre-trained models on real-time ultrasound images yielded unsatisfactory results in the classification of PCOS, primarily due to their pre-training on the ImageNet dataset. Consequently, there is a need for the creation of a specialized model tailored specifically for PCOS classification using ultrasound images. In the current study, we proposed a CNN-based custom network named F-Net for classification. F-Net is constructed with the convolution layers, pooling layers, and batch normalization followed by the activation function. The convolution layer processes the input image with dimensions 224x224x3. Batch normalization is integrated after each mini batch to ensure stable training of the deep neural network, reducing the required number of training epochs. The max pooling layer down-samples the feature map by determining the maximum value for patches in feature maps. Additionally, the average pooling layer computes the average values of features. F-Net consists of 7 convolution layers with a filter size of 1x1 and a stride of 1. It incorporates a maximum pooling layer with a stride of 1, batch normalization, one average pooling layer, and three FC layers with a SoftMax activation function. After three convolution layers, batch normalization and max pooling operations are performed in the construction of F-Net. Fig 3 represents the F-Net architecture for the classification of normal and PCOS.

## Result

### Object detection

**Fig 4:**
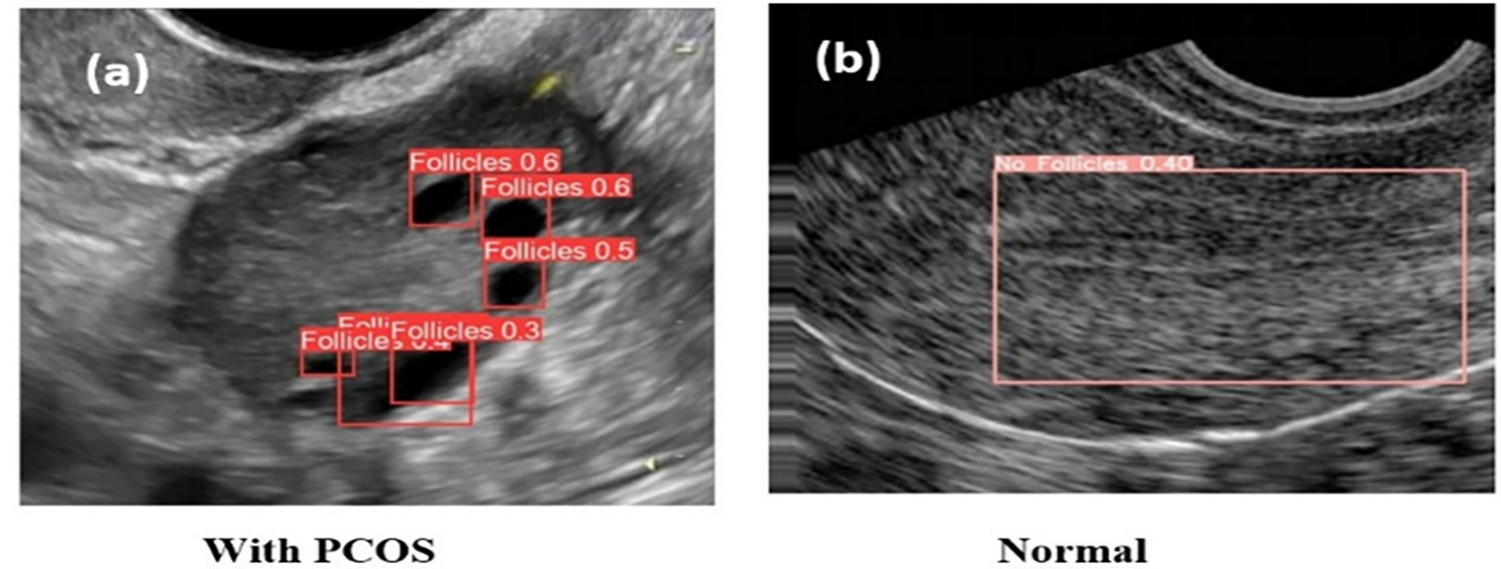
YOLOv8 output (a) follicles detected, (b) no follicles detected.

The CNN serves as the foundational architecture for the YOLOv8 model. The dataset employed in this study comprises 50 ultrasound images with follicles and 50 images without follicles. To create the training and testing datasets, a percentage split of 80–20% was applied. For the task of follicle detection, the study utilized a batch size of 32 and employed SGD optimization with a learning rate of 0.001. The training process involved the entire dataset and was executed for 20 epochs. The YOLOv8 model processed input images with dimensions of 640x640x3 for follicle detection. As depicted in Fig 4, the FC layer of the model made predictions for the follicles, providing bounding box coordinates. These bounding box values were stored in a text file format and saved within the corresponding images. It’s important to note that YOLOv8 utilizes anchor-free detection, predicting multiple follicles based on their centres. The results of follicle detection using YOLOv8 are illustrated in Fig 4. Specifically, Fig 4A showcases the detection of follicles in PCOS images, while Fig 4B demonstrates the absence of follicles in normal images. The running time of YOLOv8 model in follicle detection is 18.3ms duration.

Risk analysis in object detection is explained as follows: Detecting multiple objects in a single image, especially with various sizes and shapes, presents the most challenging aspect of object detection methods. To assess the algorithm’s effectiveness, we utilized Mean Average Precision (mAP), a widely recognized performance metric. In the context of YOLOv8, mAP is used to evaluate the object detection model. mAP involves comparing the ground-truth bounding box with the detected bounding box. In our case, the proposed YOLOv8 model, trained on our custom PCOS dataset, achieved a mAP of 80%, indicating its suitability for PCOS detection. YOLOv8’s object detection model encompasses both localization and classification. Localization involves pinpointing the coordinates of the object’s bounding box, while classification identifies whether an object exists in the image and specifies its class (e.g., PCOS or Normal). During observations, it was noted that enhancing the IoU threshold to 50% improved performances, resulting in an average precision of over 80%. However, this elevated threshold led to multiple predictions of follicles within a single image.

### Segmentation

**Fig 5:**
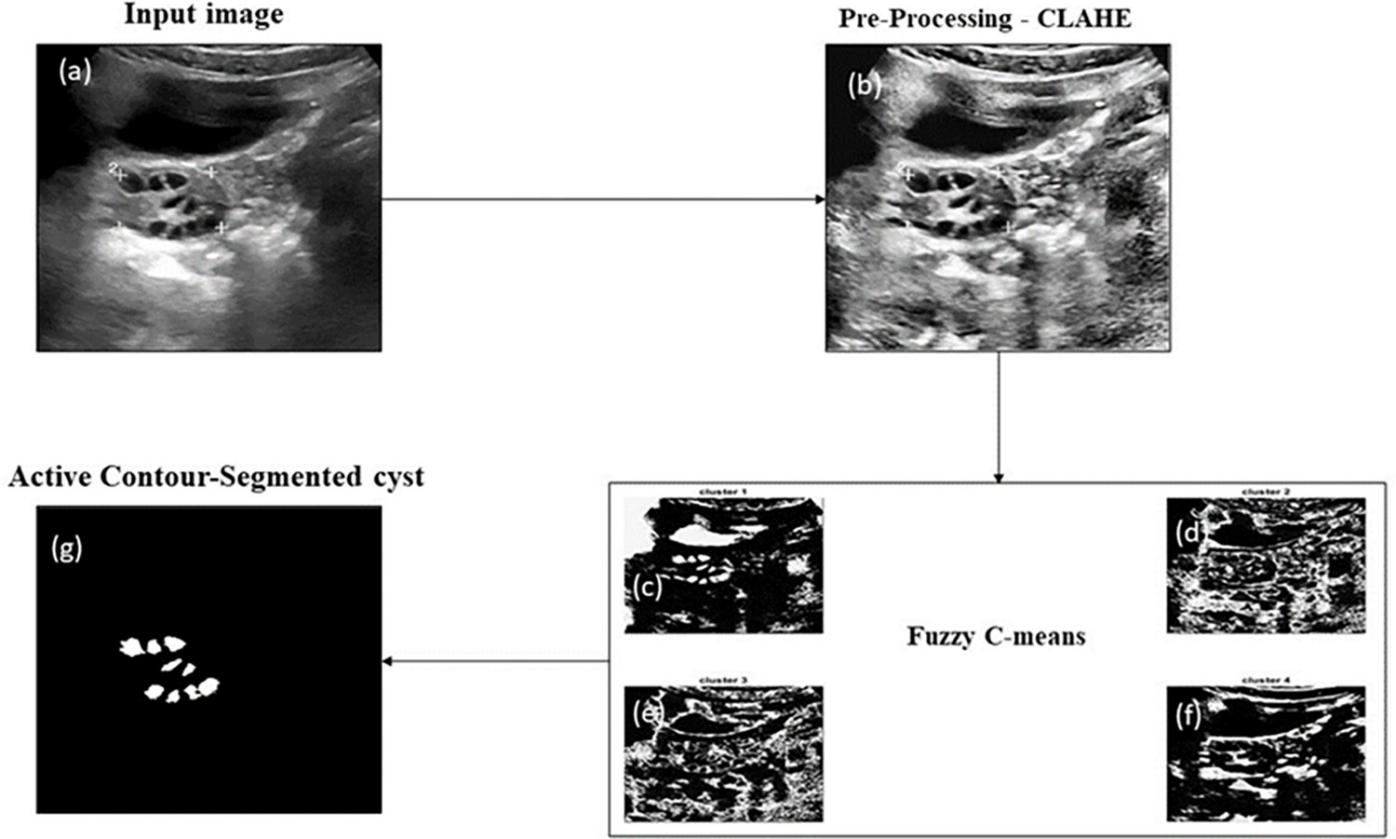
Follicle Segmentation process in ultrasound images of PCOS a) original Ultrasound image of PCOS, b) noise removed using CLAHE operation, c) cluster 1 represents edges in the cyst, d) cluster 2 shows segmented fluids with some follicles edges, e) cluster 3 indicates segmented follicles, f) cluster 4 depicts the background fluids, g) displays the final segmented follicles.

**Fig 6:**
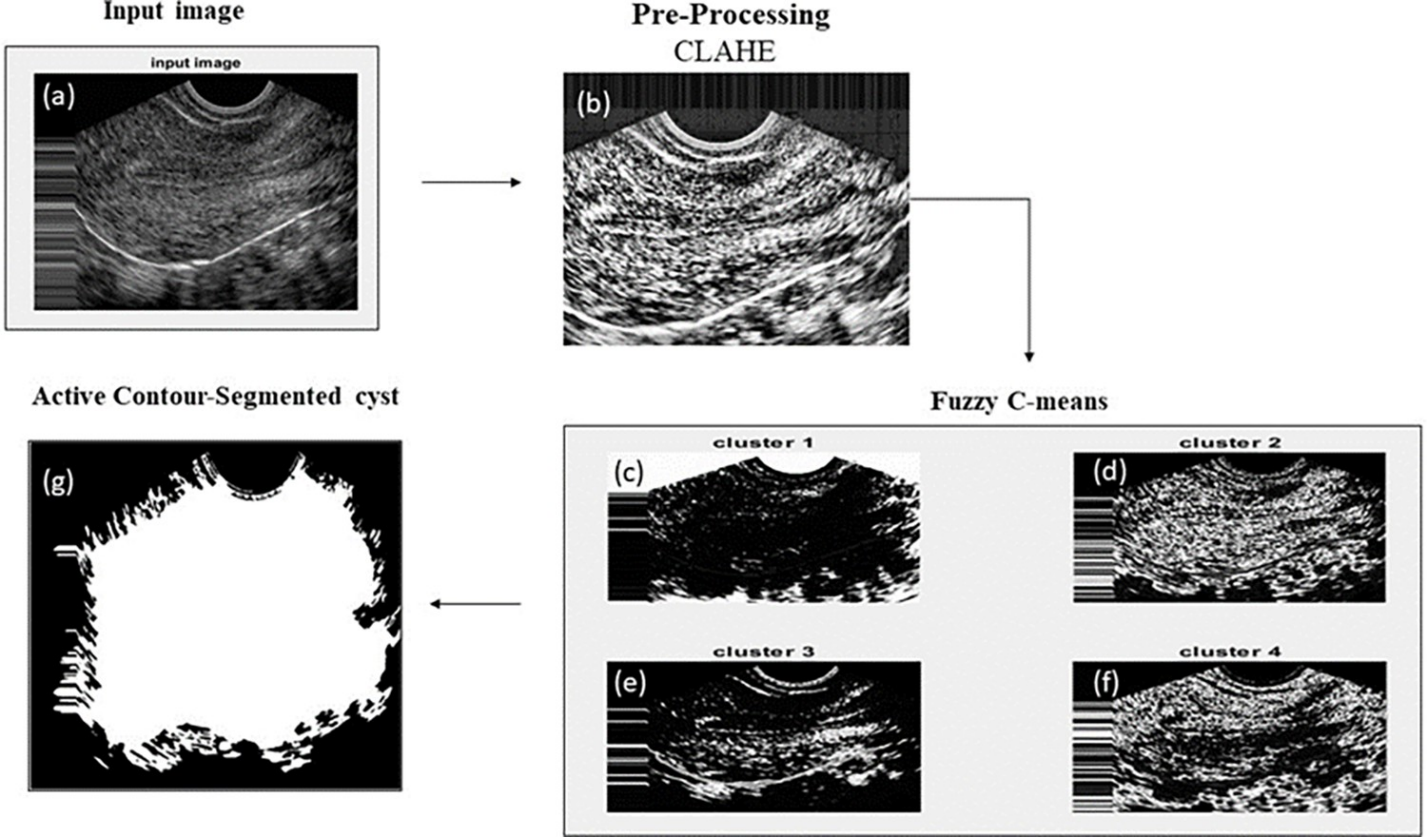
Image segmented without PCOS a) original Ultrasound image without follicles, b) noise removed using CLAHE operation, c) cluster 1 represents edges of the image, d) cluster 2 shows region of interest, e) cluster 3 indicates the surrounding region of the image, f) cluster 4 represents the fluid region of the image, g) displays final segmented image.

**Fig 7:**
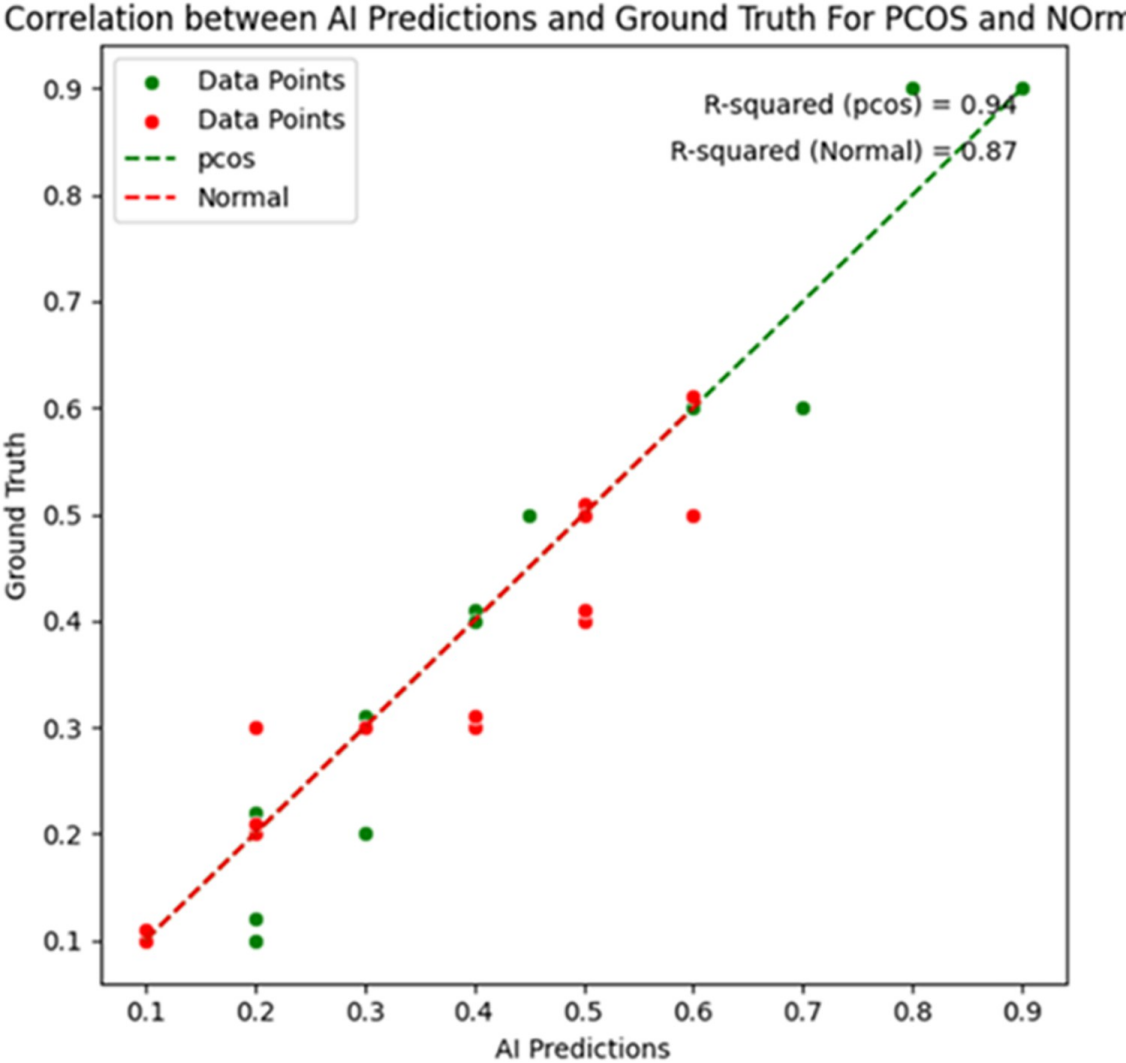
Correlation plot between AI based segmentation and ground truth segmentation.

The segmentation of the follicle’s region employs a hybrid algorithm known as the FCM based active contour method. In Fig 5, the segmented follicles from a PCOS ultrasound image are displayed, while Fig 6 depicts the segmentation of a normal image. The input ultrasound images contain considerable noise before segmentation, necessitating pre-processing steps. Initially, the images are converted to grayscale to ensure uniform contrast across all regions, and Gaussian filtering is applied to reduce image noise. The FCM method is then employed to segment the image into four clusters, each providing distinct information. Cluster one identifies the brighter region, the second cluster represents the background, the third cluster outlines the edges of the follicles, and the fourth cluster highlights the overall edges. For the active contour segmentation process, the third cluster is selected from the four clusters, yielding the final segmentation result for the follicles. Similarly, abdominal ultrasound images without follicles are segmented to differentiate between pixels in regions with and without follicles. In the segmentation phase, none of the four clusters exhibit any follicle discoveries. Using the ROI, a specific region is selected, and features are extracted from that region. The average distance between AI and Ground Truth (GT) segmentation is calculated in the form of mean ± standard deviation for PCOS (0.02±0.01) mm and Normal (0.01±0.01) mm. Fig 7 indicates the correlation plot between AI based and GT segmentation. The correlation coefficient (r) measures the proximity between two points. The r value for PCOS and Normal is 0.94 and 0.87, respectively. The observed r values indicate precise correlation between the GT and AI bounding boxes.

### Feature extraction

Ten numerical features were extracted using the GLCM algorithm, manually obtained from the segmented cyst before classification. Statistical characteristics including contrast, energy, homogeneity, correlation, entropy, skewness, and kurtosis were extracted from both normal and abnormal ultrasound images with PCOS. Each feature provides distinct pixel information about the segmented region, and these features are tabulated in Table 1 in the form of mean ± standard deviation. In PCOS images, the energy level is observed to be 35% (dataset1) and 37.3%(dataset2) higher compared to normal images, indicating a greater uniformity in the region. Homogeneity is also 18% (dataset1) and 14% (dataset2) higher in PCOS, suggesting closer proximity of data points and greater similarity of characteristics within the region. The correlation exhibits a 1.2% (dataset1) and 3.65% (dataset2) increase in PCOS compared to normal images. Additionally, skewness is notably 60%(dataset1) and 87% (dataset2) higher in PCOS images, reflecting higher peak levels of pixel values. Kurtosis experiences a 22.4%(dataset1) and 44% (dataset2) increase in PCOS images. In contrast, entropy is 47.4%(dataset1) and 59.5% (dataset2) higher in normal images compared to PCOS images, indicating a greater irregularity in the latter. The contrast value in normal images is 80% (dataset1) and 88%(dataset2) higher than that in PCOS images, potentially attributed to the wider region covered by the normal image compared to the follicles.

**Table 1:** GLCM feature extraction from segmented PCOS and normal images.

Features	Dataset1 (N = 100)				Dataset2 (N = 200)			
	PCOS (N = 50)	Normal (N = 50)	% difference	P-Value	PCOS (N = 100)	Normal (N = 100)	% difference	P-Value
**Mean**	22.32±17.57	62.78±45.69	64.4	2.646E-06	7.55±8.9	111.71±29.89	93.2	9.137E-57
**Variance**	23.80±18.02	65.52±43.85	663.6	8.596E-07	9.11±9.53	113.42±25.91	92	6.283E-62
**Std dev**	4.53±1.86	7.68±2.59	41	2.009E-07	2.72±1.31	10.57±1.29	73.7	9.368E-68
**Contrast**	0.44±0.29	2.21±0.87	80	1.859E-18	0.44±0.33	3.69±1.14	88	7.03E-47
**Energy**	0.81±0.13	0.6±0.15	35	1.652E-09	0.92±0.07	0.67±0.06	37.3	4.59E-78
**Homogeneity**	0.97±0.01	0.82±0.06	18	1.329E-24	0.98±0.01	0.86±0.04	14	9.77E-47
**Correlation**	0.83±0.09	0.82±0.04	1.2	0.615	0.79±0.2	0.82±0.09	3.65	7.78E-08
**Entropy**	0.62±0.32	1.18±0.38	47.4	2.087E-10	0.68±0.21	1.68±0.23	59.5	4.11E-68
**Skewness**	4.34±3.41	1.75±1.21	60	2.0708E-05	3.46±0.2	1.85±0.23	87	9.59E-33
**Kurtosis**	31.35±6.85	25.61±6.09	22.4	0.007	35.25±0621	24.45±5.01	44	5.37E-19

### Machine learning classification

In this proposed study, three different ML classifiers such as k-star, SGD, and RF were employed for the detection of PCOS. The study involved analysing and evaluating the performance of these classifiers to identify the most suitable one. The confusion matrix for each classifier provided insights into their performance. Performance measures, including accuracy, precision, and sensitivity, were calculated using parameters such as true positive (TP), false positive (FP), false negative (FN), and true negative (TN). Additionally, a receiver operator characteristics curve (ROC) was plotted based on TP and FP rates, and the classifier’s area was examined using the area under the curve (AUC). The results revealed that the SGD classifier surpassed the performance of other ML classifiers, attaining an accuracy of 90% for dataset1 and 98% in dataset2 in PCOS detection. It exhibited a sensitivity of 93% for dataset1 and 97% for dataset2 and specificity of 87% for dataset1 and 98% for dataset2 in distinguishing between normal and PCOS cases, respectively. The confusion matrix for the various ML classifiers for PCOS classification is given in Table 2. In the context of the classification performance evaluation, TP represents the number of PCOS images correctly classified as PCOS, FP shows the number of normal images incorrectly categorized as PCOS, TN indicates the count of normal images correctly classified as normal, and FN depicts the count of normal images incorrectly grouped as PCOS.

**Table 2:** Confusion matrix for the three different ML classifiers for PCOS detection for dataset1 (N = 100) and dataset 2 (N = 200).

Dataset1					Dataset2				
**RF Classifier**									
**N = 100**	Predicted Positive (PCOS)	Predicted Negative (Normal)	Percentage %		N = 200	Predicted Positive (PCOS)	Predicted Negative (Normal)	Percentage %	
**Actual Positive (PCOS)**	(TP)	(FN)	Specificity	Precision	Actual Positive (PCOS)	(TP)	(FN)	Specificity	Precision
	45	5	89	90		97	3	97	97
**Actual Negative (Normal)**	(FP)	(TN)	Sensitivity	F-1 score	Actual Negative (Normal)	(FP)	(TN)	Sensitivity	F-1 score
	9	41	83	86		2	98	98	97
**Accuracy = 86%**					Accuracy = 97%				
**K-star Classifier**									
**Actual Positive (PCOS)**	(TP)	(FN)	Specificity	Precision	Actual Positive (PCOS)	(TP)	(FN)	Specificity	Precision
	42	8	83	84		97	3	97	97
**Actual Negative (Normal)**	(FP)	(TN)	Sensitivity	F-1 score	Actual Negative (Normal)	(FP)	(TN)	Sensitivity	F-1 score
	10	40	80	82		1	99	98	98
**Accuracy = 82%**					Accuracy = 98%				
**SGD Classifier**									
**Actual Positive (PCOS)**	(TP)	(FN)	Specificity	Precision	Actual Positive (PCOS)	(TP)	(FN)	Specificity	Precision
	43	7	87	86		99	1	98	99
**Actual Negative (Normal)**	(FP)	(TN)	Sensitivity	F-1 score	Actual Negative (Normal)	(FP)	(TN)	Sensitivity	F-1 score
	3	47	93	89		3	97	97	98
**Accuracy = 90%**					Accuracy = 98%				

### Deep learning classification

In this study, the classification of PCOS and normal images was carried out using three different pre-trained models as follows: MobileNet, DenseNet121, and ResNet152V2. The training of these CNN models spanned ten epochs, with all networks utilizing the SGD optimizer and a learning rate of 0.001. Initially, the dataset was divided into a training set (80%) and a testing set (20%). Subsequently, data augmentation using the GAN technique expanded the dataset from 80 to 1000 instances, comprising 40 normal and 40 PCOS cases. From these, 70% (560 images) were designated for training, and 30% (240 images) for validation. This consistent data splitting approach was applied to both the three pre-trained classifiers and the custom (F-Net) classifier. The MobileNet exhibited an accuracy rate as 85% dataset1 and 96% for dataset2. In contrast, DenseNet121 and ResNet152V2 achieved identical accuracy rates of 90% and accompanied by corresponding AUC values of 0.99 for dataset1. But achieved an accuracy of 95% for ResNet152v2 and 92.5% for DenseNet121 for dataset2. When assessing the overall performance, MobileNet used for dataset2 demonstrated robust capabilities in effectively classifying PCOS and non-PCOS cases.

The custom model, F-Net, underwent training for ten epochs using an SGD optimizer with 0.001 as learning rate. F-Net differs from other models as it is constructed with fewer convolution layers, employing a filter size of 1x1. Impressively, it achieved the highest accuracy among all models, reaching 95% for dataset1 and 97.5% for dataset2, as given in Table 3. The confusion matrix of the F-Net classifier, depicting testing accuracy, is detailed in Table 4. To incorporate detailed analysis of False positives/Negatives, False discovery rate and false omission rate has been calculated for F-Net model. The false detection rate was obtained as 9.09% for dataset 1 and 4.76% for dataset2 and false omission rate is 0% for both the datasets.

**Table 3:** Performance metrics of various pre-trained and custom model.

Description	Pre-trained model	Accuracy	Precision	recall	F1
Dataset1	MobileNet	85%	0.77	1.00	0.87
	ResNet152v2	90%	0.83	1.00	0.91
	DenseNet121	90%	0.83	1.00	0.91
	Custom F-Net	95%	0.91	1.00	0.95
Dataset 2	MobileNet	96%	0.97	0.96	0.96
	ResNet152v2	95%	0.95	0.95	0.95
	DenseNet121	92.5%	0.93	0.93	0.92

**Table 4:** Confusion matrix for custom F-Net classifier.

**F-Net Classifier- Dataset1**					
N = 20	Predicted Positive (PCOS)		Predicted Negative (Normal)		%
Actual Positive	(TP)		(FN)		Specificity
(PCOS)	10		0		100
Actual Negative	(FP)		(TN)		Sensitivity
(Normal)	1		9		90
Accuracy = 95%					
**F-Net Classifier- Dataset2**					
N = 40		Predicted Positive (PCOS)		Predicted Negative (Normal)	%
Actual Positive		(TP)		(FN)	Specificity
(PCOS)		20		0	100
Actual Negative		(FP)		(TN)	Sensitivity
(Normal)		1		19	95
Accuracy = 97.5%					

**Fig 8:**
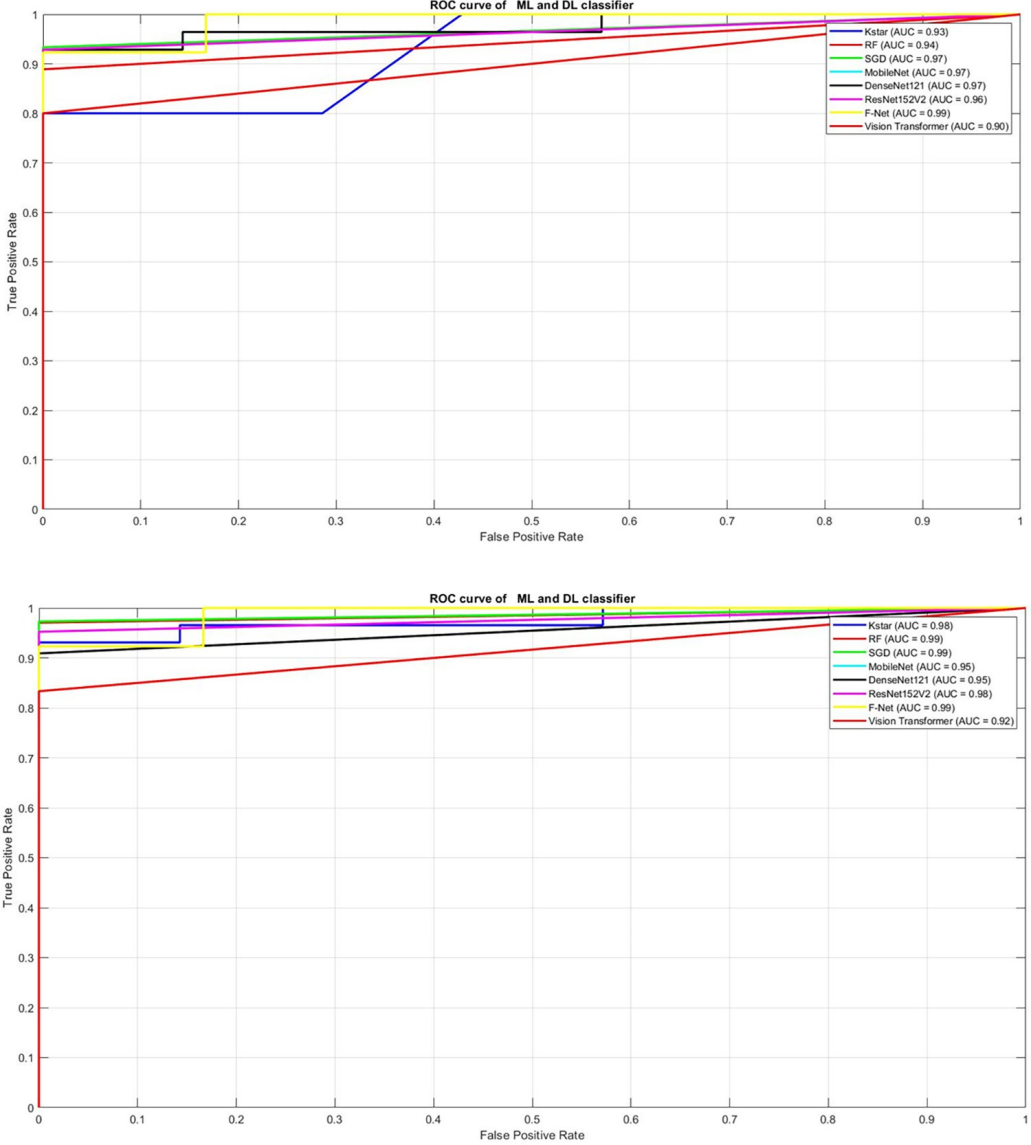
a. ROC curve for Machine learning and Deep learning classifier for Dataset1. b. ROC curve for Machine learning and Deep learning classifier for Dataset2.

Furthermore, Fig 8 displays the ROC curve for both ML and DL classifiers in PCOS identification. The AUC value of the curve acts as a reliable measure of the classifier’s performance, with values closer to 1.0 considered excellent in practical applications. In this study, both the SGD and RF classifiers demonstrated excellent performance, achieving AUC values of 0.97 and 0.94, respectively for dataset1 and 0.99 for both SGD and RF for dataset2. Similarly, k-star classifier exhibited a commendable AUC of 0.93 for dataset1 and 0.98 for dataset2. These results suggest that all three classifiers are effective in distinguishing between PCOS and normal images, with the RF classifier showing slightly superior performance based on the AUC metric. Notably, the F-Net classifier secured the highest AUC value of 0.99 compared to other deep learning classifiers.

In terms of computational complexity, the study provides insights into the training and testing times for various deep learning networks. Mobile Net V2 took 360 secs and 24 secs for training and testing respectively, ResNet152 utilized 600 secs for training and 6 secs for testing, and DenseNet121 required 660 secs and 10 secs for training and testing respectively. In contrast, F-Net exhibited the least timing requirements, with 180 secs and 5 secs for training and testing respectively, to execute the data. This highlights F-Net’s efficiency in terms of both training and testing times compared to the other DL models in the study.

## Discussions

This study introduces an innovative approach for the early identification of PCOS. The ML classification process involves utilizing YOLOv8 to detect cysts, followed by segmenting the identified area using a combination of the FCM and active contour techniques. Features based on GLCM are then extracted from the segmented region and employed in ML classifiers. For DL classification, the input image remains unaltered, as feature extraction is automatically conducted within the deep learning framework.

Several researchers have implemented DL techniques widely in variety of applications [39–41]. Soni et al. proposed a technique that relies on region-based segmentation for identifying follicles. The background image is separated using a watershed algorithm [42], and the segmented images are subsequently subjected to classification through CNN models. It’s crucial to highlight that this method primarily serves to indicate the seriousness of the illness, classifying it into varying degrees from low to high. On the other hand, Yilmaz et al. introduced two distinct follicle detection techniques. In their first approach, they utilized morphological operations, binarization, contrast modification, and noise filtering techniques [43]. For their second approach, k-means clustering, and morphological processes were employed for segmentation. The Canny edge detection algorithm was employed for follicle detection during the segmentation stage in both techniques. However, this approach yielded the highest false acceptance rate (FAR) and false rejection rate (FRR) among the tested methods. Notably, it was observed that the k-means clustering method produced more accurate results in the second technique.

Mandal et al. introduced an automated segmentation technique for follicle segmentation from ultrasound images, utilizing a k-means clustering algorithm[44]. Before applying the clustering approach, contrast enhancement via histogram equalization was performed on the images. Subsequently, morphological operations were applied to enhance the distinctiveness of the clustered segments. The suggested method achieved a classification rate of 84.6% and a precision of 90.9%. It’s important to note that in the literature mentioned, segmentation was conducted without direct follicle detection. In our proposed work, we not only detect the follicles but also determine their follicle counts by means object detection. Additionally, the literature predominantly relied on conventional segmentation methods. In contrast, our proposed approach combines the FCM and active contour techniques, resulting in a fusion of two methods.

Thakre et al., identified PCOS by employing a combination of minimal and optimal parameters in association with five ML classifiers [45]. Notably, among the five classifiers examined in their research. RF exhibited the utmost accuracy, reaching an impressive success rate of 90.9%. Nasim and their team employed ten different ML techniques to classify PCOS, utilizing a set of 39 features [46]. They employed Chi-squared (CS) feature selection method to identify the most predominant features. Interestingly, in their investigation, Gaussian Naïve Bayes emerged as the top-performing method, achieving a remarkable accuracy rate of 100% while requiring less computational time.

Alamoudi et al., devised two distinct approaches for diagnosing PCOS [47]: (i) The first method focuses on identifying patients with PCOS. In their approach, DL architectures are employed to extract automated features from images and subsequently categorize them. (ii) In the second method, baseline variables such as BMI, weight, age, in addition to ultrasound images, are utilized to detect PCOS. This is achieved through a data fusion technique combined with DL models. Two joint fusion models, namely Joint Fusion Type I and Fusion Type II, were developed and evaluated. In the realm of DL, the Inception V3 model achieved a precision rate of 69.57%, specificity of 87.98%, sensitivity of 76.19%, and an overall accuracy of 84.81%. Upon analysing the two-fusion models, Joint Fusion Type I achieved an accuracy of 82.46% when using the Mobile Net model, while Joint Fusion Type II attained an accuracy rate of 77.19% when employing the VGG16 model.

Furthermore, this study introduced a unique custom CNN model named F-Net, which, is a novel development. In this research, we compared ML classifiers such as *k-*star, SGD, and RF with DL classifiers including Mobile Net, ResNet152v2, DenseNet121, and our custom F-Net. Remarkably, our custom DL model, F-Net, achieved the highest accuracy of 95% for dataset1 and 97.5% for dataset2 when classifying PCOS and normal images, surpassing the performance of both ML, pre-trained models, and Vision transformer. Table 5 represents the performance comparison of existing literature related to machine learning and deep learning techniques in PCOS detection.

**Table 5:** Performance comparison of existing literature related to machine learning and deep learning techniques in PCOS detection.

	Author	Method	Accuracy
Machine learning techniques	Dewi et al 2018 [48]	Competitive Neural network	80.84%
	Rachana et al 2021 [15]	Naïve bayes	82.05%
		SVM	84.26%
		Decision tree	87.17%
	Suha et al 2022 [1]	SVM	80.7%
		NB	76%
		RF	88.3%
		KNN	84.9%
	Alagarsamy et al 2023 [49]	SVM	93.9%
		NB	90.42%
		KNN	88.24%
	Proposed work-Dataset1	RF	86%
		K-star	82%
		SGD	90%
	Proposed work-Dataset2	RF	97%
		K-star	98%
		SGD	98%
Deep learning techniques	Alsibai et al 2023 [50]	Dense Net 201 model	62.92%
	Gulhan et al 2023 [51]	CNN	77.81%
		SqueezeNet	75.54%
	Alamoudi et al 2023 [47]	Inception V3	84.81%
		MobileNet	69.62%
		ResNet152V2	72.15%
		DenseNet121	68.35%
	Proposed work Dataset1	MobileNet	85%
		ResNet152V2	90%
		DenseNet121	90%
		Custom F-Net	95%
		Vision Transformer	78.12%
	Proposed work Dataset2	MobileNet,	97.5%
		ResNet152V2	95%
		DenseNet121	92.5%
		Custom F-Net	97.5%
		Vision Transformer	90%

The study acknowledges several limitations that warrant consideration. The study may lack clinical validation of the proposed automated system in real-world healthcare settings. While achieving high classification accuracy is a significant milestone, the effectiveness of the system in improving patient outcomes or clinical workflow efficiency needs to be validated through prospective clinical studies. Most of the deep learning models are black box in nature, unable to predict how they arrive the decisions. These models required frequent updates to adapt to new data. Hence posing challenges for long-term maintenance and sustainability.

The scalability and practical deployment of an automated system in clinical settings can indeed present challenges due to several factors. Firstly, clinical environments often vary widely in terms of size, resources, and patient population, making it difficult to implement a one-size-fits-all solution. Additionally, integrating an AI-based system into existing healthcare infrastructure requires careful planning and coordination to ensure compatibility and seamless operation within established workflows.

Ensuring compliance with regulatory standards is crucial in healthcare, where patient safety and confidentiality are paramount. AI systems in clinical settings must adhere to certain regulations to safeguard patient data and maintain trust among healthcare professionals and patients alike.

Addressing technical hurdles related to data privacy and security is another essential consideration. Healthcare data is highly sensitive and subject to strict privacy regulations, requiring robust measures to protect against unauthorized access, breaches, and misuse. Implementing encryption, access controls, and regular security audits are vital steps in mitigating these risks and ensuring the integrity and confidentiality of patient information.

In summary, the challenges associated with deploying an automated system in clinical settings stem from the need to address scalability, integrate with existing infrastructure, comply with regulatory standards, and safeguard data privacy and security. By carefully considering and actively managing these factors, healthcare organizations can successfully leverage AI technology to improve patient care and operational efficiency.

As a future work, there is potential for extending the research to develop a web application system capable of not only detecting the presence of PCOS, but also accurately assessing its stage by considering the factors such as cost-effectiveness, ease of integration into existing clinical workflows, and acceptance by healthcare providers and patients.

In conclusion, the proposed study developed a computerized framework that automatically detects Follicles in an ultrasound image. Our research focused on object detection combined with ML and DL models. YOLOv8 object detection demonstrates precise detection of multiple follicle regions within a single image, all achieved in remarkably reduced processing time. Following the follicle detection phase, the ROI was segmented using FCM based active contour algorithm. Subsequently, GLCM features were extracted, and machine learning methods were employed for classification. The SGD classifier delivered the most favorable results in terms of accuracy, precision, and sensitivity, among the four classifiers used in the study. It achieved an impressive accuracy rate of 90% for dataset1 and 98% for dataset2 for distinguishing between PCOS and non-PCOS cases. However, the custom F-Net CNN model surpassed all other classifiers in the study, achieving an exceptional accuracy rate of 95%. For datset1 and 97.5% for dataset2. Furthermore, the AUC value for F-Net was attained at 0.99, indicating a high level of performance for both the datasets. Consequently, our custom model demonstrates significant effectiveness in the classification of PCOS using ultrasound images.

## Supporting information

S1 Fig Architecture of YOLOv8 for follicle detection in PCOS. (TIF)
