# Supplementary figures and images for: F-Net: Follicles Net an efficient tool for the diagnosis of polycystic ovarian syndrome using deep learning techniques
Source: PLoS One. 2024 Aug 15;19(8):e0307571. doi: 10.1371/journal.pone.0307571 (PMC11326594; doi:10.1371/journal.pone.0307571)

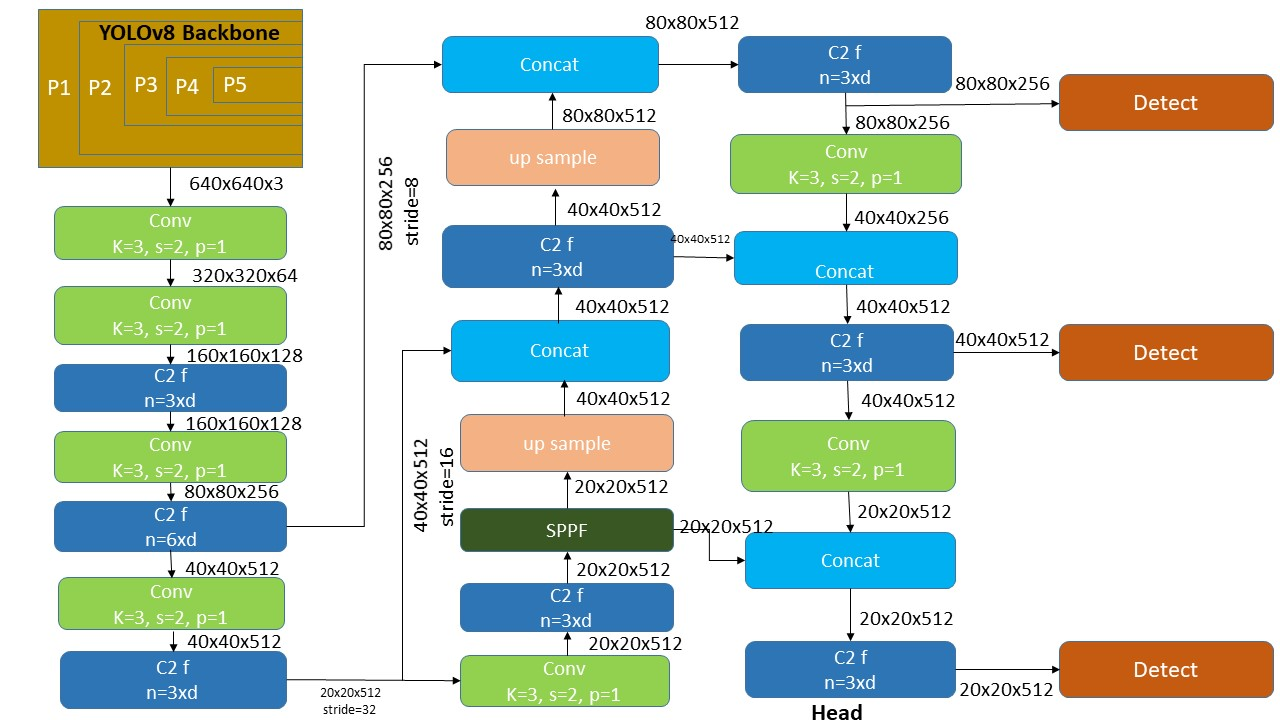

Supplement: S1 Fig — (TIF) [file pone.0307571.s001.tif]
